# Readability, Reliability, and Quality Analysis of Internet-Based Patient Education Materials and Large Language Models on Meniere’s Disease

**DOI:** 10.1177/19160216251360651

**Published:** 2025-08-08

**Authors:** Salahaldin Alamleh, Dorsa Mavedatnia, Gizelle Francis, Trung Le, Joel Davies, Vincent Lin, John J.W. Lee

**Affiliations:** 1 Temerty Faculty of Medicine, University of Toronto, Toronto, ON, Canada; 2 Department of Otolaryngology—Head and Neck Surgery, University of Toronto, Toronto, ON, Canada; 3 Faculty of Medicine, Dalhousie University, Halifax, NS, Canada; 4 Biological Sciences Platform, Hurvitz Brain Sciences Program, Sunnybrook Research Institute, Toronto, ON, Canada; 5 Department of Otolaryngology—Head and Neck Surgery, Sinai Health System, University of Toronto, Toronto, ON, Canada; 6 Evaluative Clinical Sciences Platform, Sunnybrook Research Institute, Toronto, ON, Canada; 7 Department of Otolaryngology—Head and Neck Surgery, Sunnybrook Health Sciences Centre, Toronto, ON, Canada

**Keywords:** Meniere’s disease, vertigo, artificial intelligence, medical education, quality of life

## Abstract

**Importance:**

Online patient education materials (PEMs) and large language model (LLM) outputs can provide critical health information for patients, yet their readability, quality, and reliability remain unclear for Meniere’s disease.

**Objective:**

To assess the readability, quality, and reliability of online PEMs and LLM-generated outputs on Meniere’s disease.

**Design:**

Cross-sectional study.

**Setting:**

PEMs were identified from the first 40 Google Search results based on inclusion criteria. LLM outputs were extracted from unique interactions with ChatGPT and Google Gemini.

**Participants:**

Thirty-one PEMs met inclusion criteria. LLM outputs were obtained from 3 unique interactions each with ChatGPT and Google Gemini.

**Intervention:**

Readability was assessed using 5 validated formulas [Flesch Reading Ease (FRE), Flesch Kincaid Grade Level (FKGL), Gunning-Fog Index, Coleman-Liau Index, and Simple Measure of Gobbledygook Index]. Quality and reliability were assessed by 2 independent raters using the DISCERN tool.

**Main Outcome Measures:**

Readability was assessed for adherence to the American Medical Association’s (AMA) sixth-grade reading level guideline. Source reliability, as well as the completeness, accuracy, and clarity of treatment-related information, was evaluated using the DISCERN tool.

**Results:**

The most common PEM source type was academic institutions (32.2%), while the majority of PEMs (61.3%) originated from the United States. The mean FRE score for PEMs corresponded to a 10th- to 12th-grade reading level, whereas ChatGPT and Google Gemini outputs were classified at post-graduate and college reading levels, respectively. Only 16.1% of PEMs met the AMA’s sixth-grade readability recommendation using the FKGL readability index, and no LLM outputs achieved this standard. Overall DISCERN scores categorized PEMs and ChatGPT outputs as “poor quality,” while Google Gemini outputs were rated “fair quality.” No significant differences were found in readability or DISCERN scores across PEM source types. Additionally, no significant correlation was identified between PEM readability, quality, and reliability scores.

**Conclusions:**

Online PEMs and LLM-generated outputs on Meniere’s disease do not meet AMA readability standards and are generally of poor quality and reliability.

**Relevance:**

Future PEMs should prioritize improved readability while maintaining high-quality, reliable information to better support patient decision-making for patients with Meniere’s disease.

## Graphical abstract

**Graphical Abstract:**
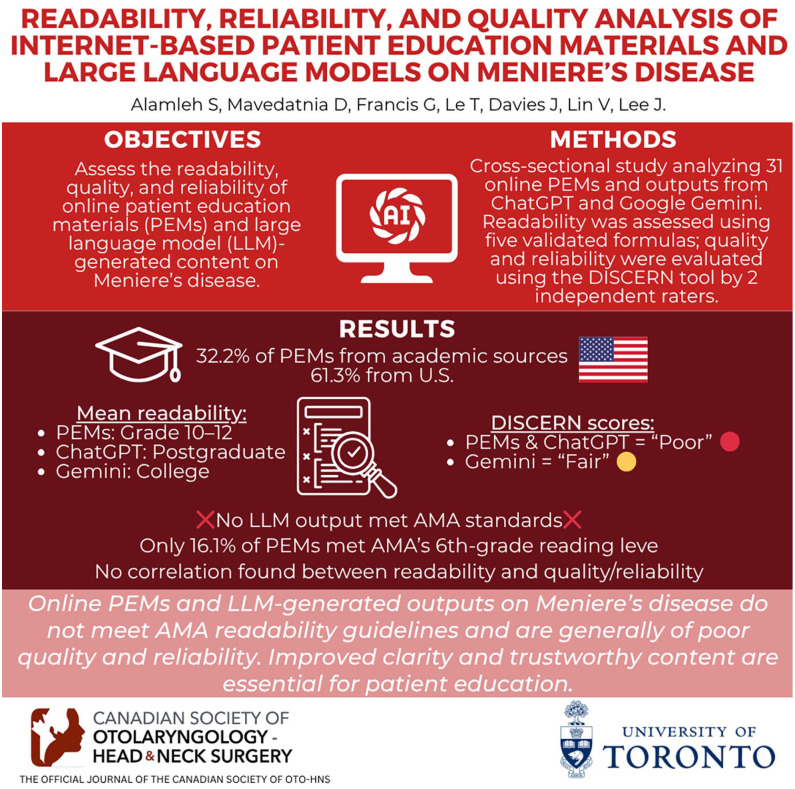

## Key Messages

1. Online patient education materials (PEMs) and large language model (LLM) outputs (ChatGPT, Google Gemini) on Meniere’s disease exceed the American Medical Association’s recommended readability guidelines, making them difficult for patients to understand.
2. The quality and reliability of online PEMs and LLM outputs (ChatGPT, Google Gemini) are generally poor, with ChatGPT scoring lowest.
3. Future online PEMs and LLM algorithms should prioritize both readability and quality to support informed decision-making for patients with Meniere’s disease.

## Introduction

Meniere’s disease is a chronic inner ear disorder characterized by recurrent episodes of vertigo, fluctuating sensorineural hearing loss, and aural symptoms such as tinnitus, ear fullness, and pressure. The etiology of Meniere’s disease remains unknown, though endolymphatic hydrops hypothesized to play a role.^1^ Sequalae of Meniere’s disease include bilateral involvement, irreversible hearing loss, and debilitating drop attacks resulting in significant impacts on quality of life.^1-3^ Online patient education materials (PEMs) are a major source of information for patients, highlighting the reliance on the Internet for healthcare-related inquiries. Patient education is crucial in fostering active participation in healthcare decisions, leading to improved outcomes, higher patient satisfaction, and reduced healthcare costs.^4^

Data from the United States and Europe indicate that a substantial portion of the general adult population, ranging from 45% to 79%, actively seeks health-related information online.^5-7^ Similarly, the emergence of large language models (LLMs), such as ChatGPT and Google Gemini, has led to a growing number of patients utilizing these technologies for medical information due to their widespread availability and user-friendly interface.^8,9^ While LLMs offer a new avenue for patients to obtain insight into their health conditions, their accuracy and reliability necessitate further evaluation.

The American Academy of Otolaryngology—Head and Neck Surgery (AAO-HNS) clinical guidelines for Meniere’s disease published in April 2020 underscores the necessity of comprehensive patient education, which should include information on the condition’s natural progression, symptom management strategies, available treatment options, and expected outcomes.^10^ Furthermore, guidelines established by the American Medical Association (AMA) state that educational material aimed at patients should be presented at a reading level equivalent to or below the sixth grade.^11-14^ Previous studies have assessed online PEMs pertaining to other otolaryngology-head and neck surgery medical conditions, such as sensorineural hearing loss, laryngectomies, and endoscopic sinus surgery, and found that PEMs were frequently written at a level above the AMA recommendations.^15-18^

The objective of this study is to assess the readability, quality, and reliability of online PEMs and LLM outputs (ChatGPT, Google Gemini) on Meniere’s disease.

## Materials and Methods

This cross-sectional study did not require Research Ethics Board approval as the data sources are openly available and do not involve identifiable or sensitive information. The methodology for this study was based on other research studies which examined the readability, reliability, and quality of PEMs in OHNS.^19,20^

### Search Strategy—Online PEMs

The Google Search engine (www.google.ca) was utilized as it has the largest market share and is the most used search engine in North America.^21^ The browser was set to incognito mode, the search history, cookies, and cached data were cleared, and the browser and location settings were disabled to minimize the influence of previous search history and location on the search results. On June 2, 2024, a search with the term “Meniere’s Disease” was done using the Google Search engine. The first 40 results were reviewed to determine eligibility based on the inclusion criteria. This approach was taken as over 90% of individuals abandon their search after viewing the first 3 pages of results, which is equivalent to ~30 search results.^22^ Included online PEMs were categorized by country of origin and type, including academic institution, government website, medical information website, professional organization, and miscellaneous.

All the results were reviewed on the same day that the search was conducted. The first 40 search results that were PEMs about Meniere’s disease were screened for inclusion. Websites were subsequentially excluded if they were non-English, lacked patient information about Meniere’s disease, required payment, were academic articles, directed for healthcare providers, contained only audiovisual materials, were blogs or forums, were advertisements, contained fewer than 100 words, or were duplicates.^15,16,19^

### Search Strategy—LLMs ChatGPT and Google Gemini

A list of questions was formulated based on the most commonly answered topics in the online PEMs. The questions included the following: “what is Meniere’s disease?” “how do you get Meniere’s disease?” “what are the symptoms of Meniere’s disease?” “how is Meniere’s disease diagnosed?” “how is Meniere’s disease treated?” “what are the complications of Meniere’s disease?” and “will Meniere’s disease go away?” In alignment with the methodology taken for identifying online PEMs on a browser, each question was asked sequentially into unique ChatGPT and Google Gemini sessions.^23^ To avoid memory-related bias, 3 independent sessions were conducted for each LLM, with all questions posed in each session. Responses from each session were recorded into separate text files using Microsoft Word (Microsoft Corp, Redmond, WA, USA), generating 3 files for ChatGPT and 3 for Google Gemini.

### Readability Evaluation

Included online PEMs and LLM outputs were assessed for readability. The texts were copied and pasted into a Microsoft Word file (Microsoft Corp). Elements that could skew scores, including author information, disclaimers, references, and publication dates, were removed prior to evaluation. Non-text elements such as abbreviations, decimals, paragraph breaks, colons, and semicolons were also removed, following established methodologies.^24,25^ Files were assessed using an open-access readability calculator (https://readable.com/) that utilizes 5 metrics to assess readability: Flesch Reading Ease (FRE), Flesch Kincaid Grade Level (FKGL), Gunning-Fog Index (GFI), Coleman-Liau Index (CLI), and Simple Measure of Gobbledygook Index (SMOG).^26-30^ Table 1 reports the formulas used to calculate the reading indices. The FRE scores ranged from 0 to 100 with a higher score corresponding to text, that is, easier to read (Table 2). The FKGL, GFI, CLI, and SMOG measured the academic grade level necessary to comprehend the text, with a higher grade level corresponding to text that was more difficult to read.

**Table 1.** Formulas for Readability Formulas.

Reading index	Formula
FRE	$206.835-1.015\left(\frac{\mathrm{total}\;\mathrm{words}}{\mathrm{total}\;\mathrm{sentences}}\right)-84.6\left(\frac{\mathrm{total}\;\mathrm{syllables}}{\mathrm{total}\;\mathrm{words}}\right)$
FKGL	
GFI^a^	$0.4\;\times \;\left[\left(\frac{\mathrm{words}}{\mathrm{sentences}}\right)+\left(\frac{\sigma }{\mathrm{words}}\;\times 100\right)\right]$
CLI^b,c^	(0.0588×γ)−(0.296×ω)−15.8
SMOG	$1.0430\times \sqrt{\sigma \times \frac{30}{\mathrm{number}\;\mathrm{of}\;\mathrm{sentences}}}+3.1291$

Abbreviations: CLI, Coleman-Liau Index; FKGL, Flesch Kincaid Grade Level; FRE, Flesch Reading Ease; GFI, Gunning-Fog Index; SMOG, Simple Measure of Gobbledygook Index.

a Number of words ≥3 syllables.

b Average number of letters per 100 words.

c Average number of sentences per 100 words.

**Table 2.** FRE Score With Equivalent Education Level.

FRE score	Equivalent education level
0-29	College graduate
30-49	College
50-59	10th-12th grade
60-69	Eighth to ninth grade
70-79	Seventh grade
80-89	Sixth grade
90-100	Fifth grade

Abbreviation: FRE, Flesch Reading Ease.

### Quality and Reliability Evaluation

Two raters (S.A. and G.F.) independently evaluated all online PEMs and LLM outputs, resolving discrepancies by consensus. The DISCERN instrument, a standardized tool, was used to assess reliability (Section 1), quality (Section 2), and global assessment (Section 3) through 16 questions scored on a 5-point Likert scale. Total scores range from 16 to 80, corresponding to global quality ratings (Table 3).

**Table 3.** DISCERN and Modified DISCERN Score and Corresponding Quality Level.

DISCERN score	Modified DISCERN score^a^	Quality level
64-80	30-35	Excellent
52-63	24-29	Good
41-51	18-23	Fair
30-40	12-17	Poor
<30	<12	Very poor

Abbreviations: LLMs, large language models; PEMs, patient education materials.

a Modified DISCERN score refers to the global quality score derived from and adapted Section 2 only scale for better comparison between online PEMs and LLMs.

To compare online PEMs and LLM outputs accurately, a separate Section 2 only score was calculated following the methodology employed by Cocci et al.^31^ This was done as Section 1 evaluates elements like aims and references, which are absent from LLM outputs. These scores were then converted to global quality ratings using a modified DISCERN scale (Table 3).

### Data Analysis

Descriptive statistics were utilized to summarize the results of the readability and DISCERN scores for online PEMs and LLM outputs. Kruskal-Wallis test followed by Dunn-Bonferroni post hoc tests were done to compare readability, quality, and reliability of PEMs based on source type. Inter-rater reliability for the DISCERN tool was assessed using the weighted kappa (*κ*) statistic. Pearson’s correlation coefficients (*r*) were calculated to evaluate the relationship between readability scores and DISCERN scores. A *P* value of .05 was used to define significance for all analyses. Statistical analyses were performed using RStudio software version 4.2.1 (Posit Software; PBC, Boston, MA, USA).^32^

## Results

### Online PEM Selection

**Figure 1.**
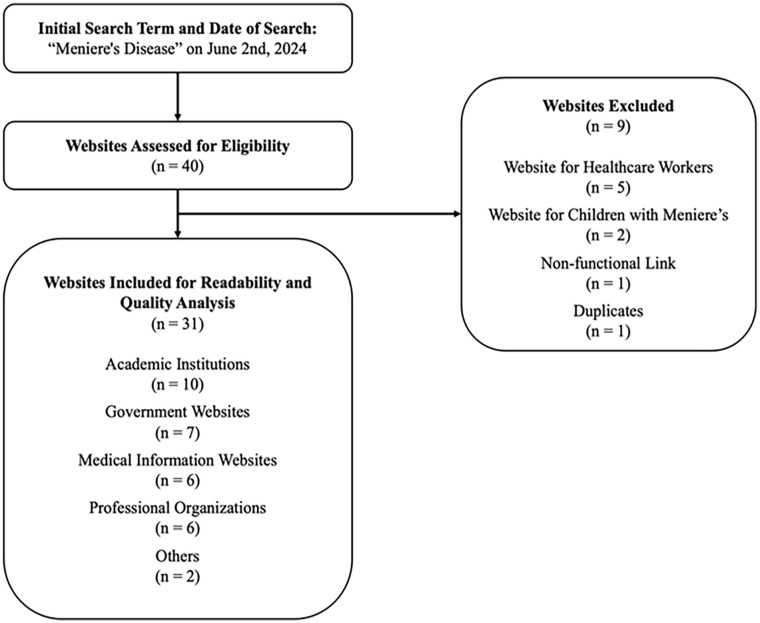
Website inclusion and exclusion criteria.

From the 40 Google Search results, 31 met the inclusion criteria. Reasons for exclusion are cited in Figure 1. Of the 31 included online PEMs, 61.3% (19/31) originated from the USA, 25.8% (8/31) from Canada, 6.5% (2/31) from the United Kingdom, and 6.5% (2/31) from Australia. The majority of online PEMs were from academic institutions (32.2%, 10/31), followed by government websites (7/31, 22.6%), medical information websites (6/31, 19.4%), and professional organizations (6/31, 19.4%).

### Readability Evaluation

The average FRE score for online PEMs was 53.3 (SD 10.4) corresponding with a 10th- to 12th-grade reading level. Only 1 PEM scored below above 80 on the FRE, corresponding with the AMA recommendation of sixth-grade reading level. The average FRE scores were 23.9 (SD 3.3) for ChatGPT and 39.8 (SD 3.0) for Google Gemini, corresponding with a post-colleague graduate and college student reading level, respectively. None of the LLM outputs received an FRE score at or below the AMA recommendation of sixth-grade reading level (FRE score of 80 or above).

**Figure 2.**
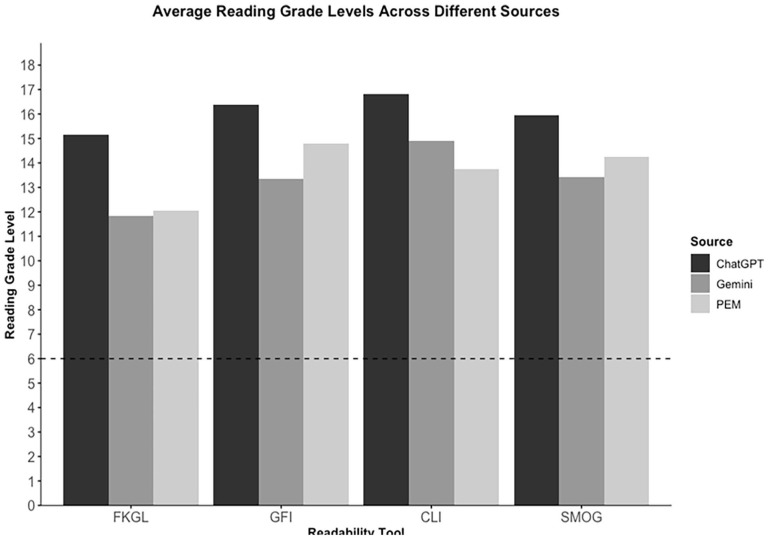
Average readability tool scores for online PEMs and LLMs. LLMs, large language models; PEMs, patient education materials. The dashed line at reading grade level “6” corresponds with the recommendation from the Association of American Medical College (AAMC) for education material geared toward patients.

Figure 2 illustrates the readability score distributions as determined by the FKGL, GFI, CLI, and SMOG. PEMs from government websites were scored the easiest to read and those originating from miscellaneous sources were scored as the most difficult to read, though this was not statistically significant. LLM outputs, on average, were more difficult to read than online PEMs, with ChatGPT having the highest reading grade level across all readability indices (Table 4). Only 16.1% (5/31) of online PEMs met the AMA’s recommendation of a grade-6 reading level with the FKGL formula. None of the online PEMs or LLM outputs met the AMA recommendations using the other readability formulas.

**Table 4.** Mean Readability Scores According to Source Type.

Readability tool, mean (SD)	Online patient education material						Large language models	
	All PEMs (n = 31)	Academic institutions (n = 10)	Government websites (n = 7)	Medical information websites (n = 6)	Professional organizations (n = 6)	Miscellaneous (n = 2)	ChatGPT (n = 3)	Google Gemini (n = 3)
FRE score	53.3 (10.4)	52.6 (6.7)	62.6 (13.4)	52.1 (11.2)	48.7 (4.8)	42.6 (8.1)	23.8 (3.3)	39.8 (3.0)
FKGL score	9.0 (1.9)	8.9 (1.3)	7.9 (2.6)	9.1 (1.9)	10.2 (1.0)	10.8 (1.8)	14.6 (0.5)	11.5 (0.6)
GFI score	10.8 (2.1)	10.4 (1.9)	9.7 (2.7)	10.6 (1.9)	12.1 (1.2)	12.7 (3.0)	16.2 (0.5)	13.0 (0.5)
CLI score	11.6 (1.6)	11.6 (1.0)	10.1 (2.2)	11.9 (1.6)	12.4 (0.8)	12.1 (0.8)	15.6 (0.3)	14.3 (0.6)
SMOG score	11.3 (1.6)	11.1 (1.3)	10.3 (2.0)	11.2 (1.3)	12.4 (1.0)	12.8 (2.0)	15.6 (0.3)	13.1 (0.4)

Abbreviations: CLI, Coleman-Liau Index; FKGL, Flesch Kincaid Grade Level; FRE, Flesch Reading Ease; GFI, Gunning-Fog Index; PEMs, patient education materials; SMOG, Simple Measure of Gobbledygook Index.

There was no significant difference in readability scores depending on the origin of the online PEMs: FRE (*P* = .18), FKGL (*P* = .21), SMOG (*P* = .18), CLI (*P* = .12), and GFI (*P* = .21).

### Quality and Reliability Analysis (DISCERN)

Average total DISCERN scores were 38.5 for online PEMs, 27.5 for ChatGPT, and 35.0 for Google Gemini, all corresponding to “poor quality” (Table 5). PEMs from medical information websites had the highest average total score (41.6, SD 4.0, “fair” quality) and those from academic institutions had the lowest average total score (36.1, SD 9.5, “poor” quality). Of the PEMs evaluated, 3% (1/31) were rated “excellent” quality, 3% (1/31) “good” quality, 36% (11/31) “fair” quality, 48% (15/31) “poor” quality, and 10% (3/31) “very poor” quality. All session outputs from ChatGPT and Gemini were rated “poor” quality. Comparing Section 2 of DISCERN, which exclusively evaluates quality, the average score was 15.5 (SD 4.1) for online PEMs, 15 for ChatGPT, and 20 for Gemini. Only 3% (1/31) of online PEMs were rated “good” quality, 16% (5/31) “fair” quality, 65% (20/31) “poor” quality, and 16% (5/31) “very poor” quality. All ChatGPT outputs (3/3) were rated “poor” quality, while all Google Gemini outputs (3/3) were rated “fair” quality.

**Table 5.** Average DISCERN Score Comparison Across Online PEMs and LLMs^a^.

Criterion	Mean PEM score (SD)	Mean ChatGPT (SD)	Mean Gemini score (SD)
Section 1: Reliability			
1. Are the aims clear?	2.0 (1.4)	1.0 (0.0)	1.0 (0.0)
2. Does it achieve its aim?	1.8 (2.2)	-	-
3. Is it relevant?	3.5 (0.7)	3.0 (0.0)	3.0 (0.0)
4. Is it clear what sources of information were used to compile the publication?	1.9 (1.2)	1.0 (0.0)	1.0 (0.0)
5. Is it balanced and unbiased?	2.3 (1.3)	1.0 (0.0)	1.0 (0.0)
6. Does it provide details of additional sources of support and information?	3.5 (0.9)	2.0 (0.0)	3.0 (0.0)
7. Is it clear when the information used or reported in the publication was produced?	1.9 (1.4)	1.0 (0.0)	1.0 (0.0)
8. Does it refer to areas of uncertainty?	3.0 (0.7)	3.0 (0.0)	3.0 (0.0)
Section 2: Quality			
9. Does it describe how each treatment works?	2.6 (1.2)	2.0 (0.0)	3.0 (0.0)
10. Does it describe the benefits of each treatment?	2.8 (1.1)	3.0 (0.0	3.0 (0.0)
11. Does it describe the risks of each treatment?	2.0 (1.1)	2.0 (0.0)	3.0 (0.0)
12. Does it describe what would happen if no treatment is used?	1.5 (0.8)	1.0 (0.0)	4.0 (1.1)
13. Does it describe how the treatment choices affect overall quality of life?	1.6 (0.8)	1.0 (0.0)	1.0 (0.0)
14. Is it clear that there may be more than 1 possible treatment choice?	3.3 (0.9)	3.0 (0.0)	4.0 (0.0)
15. Does it provide support for shared decision-making?	1.7 (1.2)	1.0 (0.0)	1.0 (0.0)
16. Overall rating of publication	2.7 (0.7)	2.5 (0.5)	3.0 (0.0)
Total Section 2 score	16.9 (4.3)	15 (0.0)	20 (0.0)
Total DISCERN score	38.5 (9.2)	27.5 (0.5)	35 (1.0)

Abbreviations: LLMs, large language models; PEMs, patient education materials.

a The mean rating represents the mean score for each question in the DISCERN instruments for all the included PEMs.

There was no significant difference in reliability (*P* = .40) or quality (*P* = .53) scores based on the source type of the online PEMs. There was no significant difference in the total DISCERN scores between the initial 3 search results and the remaining PEMs [41.2 (SD 10.2) vs 38 (SD 9.2), *P* = .65]. Additionally, no significant correlation was found between average total DISCERN score of PEMs and any of the readability indices: FRE (*P* = .57), FKGL (*P* = .33), CLI (*P* = .55), GFI (*P* = .45), and SMOG (*P* = .35).

Cohen’s weighted kappa for the total DISCERN score and Section 2 scores for online PEMs were 0.92 and 0.89, respectively, indicating excellent interrater reliability. Cohen’s weighted kappa could not be calculated for the LLMs due to limited sample size.

## Discussion

This study assessed the readability, quality, and reliability of online PEMs and LLM outputs for Meniere’s disease. Our findings suggest that online PEMs and LLM outputs demonstrate significant limitations and deficits in these areas. PEMs on Meniere’s disease are consistently written at a 10th- to 12th-grade level, while LLMs are even more complex, ranging from college to post-graduate reading levels. Both sources exceeded the recommended sixth-grade level for optimal patient understanding. Additionally, both PEMs and LLMs scored poorly in reliability and quality. These results emphasize the need for educational materials with improved readability and quality for patients with Meniere’s disease.

With widespread Internet access, patients increasingly seek online resources for health information. However, the quality and clarity of this information remain inconsistent. This is concerning, as studies highlight patient dissatisfaction with their understanding of Meniere’s disease and the negative impact of low health literacy on disease outcomes.^33,34^ The AAO-HNS highlights the critical role of patient education in managing Meniere’s disease, as seen in improved outcomes for other chronic conditions like asthma, diabetes, and cancer.^10,35^

Reading is essential for health literacy, with Americans reading at an average of an 8-grade level, at least 3 grades lower than their education level.^36^ The AMA recommends that Internet-based PEMs be developed at or below a sixth-grade reading level for accessibility. Almost all (97%) PEMs and all LLM outputs scored below 80 on the FRE scale, indicating they failed to meet this guideline and were “difficult” or “very difficult” to read. These findings are in-keeping with Cherla et al, that found 96.8% of online PEMs on endoscopic sinus surgery exceeded the sixth-grade reading level.^17^ Our results for LLM outputs correspond with Eid et al, which demonstrated that ChatGPT consistently rated as more difficult to read than online PEMs in ophthalmologic and reconstructive surgery.^37^ Given that 48% of Canadians and 36% of Americans are reported to have low literacy, it may be more difficult for this population to benefit from currently available information.^38,39^ This may result in reduced participation in medical decisions, poor adherence to recommendations, and higher risk of adverse outcomes.^19,40-42^ Previous studies have demonstrated that partnerships with libraries and multidisciplinary professionals can enhance the delivery of health information by improving readability.^43^ To enhance future PEMs on Meniere’s disease, librarians with expertise in health literacy and patient education should be involved in the writing process. Furthermore, authors should minimize the use of complex words, decrease the number of words per sentence and syllables per word, utilize numbering or bullet points, and write in active voice.^15,19,41,42^

Aside from readability, education materials must also provide reliable and evidence-based information. Using the DISCERN tool, 61% of online PEMs and LLMs received a “poor” or “very poor” global rating, indicating substantial shortcomings in reliability and quality. Although not statistically significant, PEMs from medical information websites (eg, WebMD, Healthline) had higher average DISCERN scores than those from academic institutions like Harvard University and the Cleveland Clinic. While both sources showed similar quality scores, medical information websites were more consistent in providing publication dates and references, challenging the perception that academic sources are inherently more trustworthy. This discrepancy underscores the importance of rigorously evaluating PEMs, regardless of their origin. One online PEM, originating from a professional organization focused on balance and dizziness disorders, received an “excellent” global rating, though it still exceeded the recommended sixth-grade reading level. This underscores that even high-quality and reliable PEMs can be ineffective if they are not easily understandable for the general population. Similar results were seen in Seymour et al who found that based on DISCERN scores, 63% of PEMs about cochlear implants were rated as “poor” or “very poor” quality.^18^ Similarly, Grose et al found that 42% of PEMs for septoplasties were of “poor” or “very poor” quality.^19^ This highlights the need for healthcare providers and developers to create online PEMs that are comprehensible, balanced, and unbiased.

Regarding reliability (questions 1-8), online PEMs consistently scored poorly on stating aims (mean score 2), providing references (mean score 1.9), and citing publication dates (mean score 1.9). The absence of sources and publication dates increases bias and limits access to evidence-based information, especially for chronic conditions like Meniere’s disease, where ongoing research continually introduces new treatment and management strategies.^19^ Similarly, LLMs also lacked stated aims and references, posing a significant limitation. Without references and an inability to cross-reference citations, LLMs pose a risk of hallucinations, defined as occurrences where LLMs generate plausible but false or misleading information. Additionally, LLMs are known to generate varied responses depending on context and input, potentially impacting health decisions.^44^ To improve reliability, future PEMs should outline objectives, provide references, and include revision dates. LLM developers can reduce hallucinations by implementing validation processes, continuously update models with up-to-date research, and providing transparency about the sources and their limitations.^45^ As these models evolve, enhancing their ability to deliver accurate, high-quality, and understandable content is crucial for effective patient education.

In terms of quality scores (questions 9-15), ChatGPT outputs were of the lowest quality, followed by online PEMs, and Google Gemini outputs (Table 5). Previous literature has demonstrated ChatGPT to have lower quality responses and is more difficult to comprehend when compared to information from Google searches on benign paroxysmal positional vertigo.^46^ Both PEMs and LLMs consistently scored poorly on describing how each treatment works, the benefits, risks, impact on quality of life, and providing support for shared decision-making. The lowest scoring questions for PEMs were the consequences of foregoing treatment and the impacts on treatment on quality of life. Omission of this information may not only negatively impact the informed consent process, but also bias patients when making treatment decisions.^14^ Given that Meniere’s disease is a chronic condition with multiple treatment options and no established gold standard, online PEMs that comprehensively review available treatment options are essential.^47^ Future PEMs should ensure to clearly outline the various treatment options and address the relevant risks and benefits, including the potential effects of foregoing treatment.

The present study is a novel comparative analysis of online PEMs and LLM outputs pertaining to Meniere’s disease, though it is not without limitations. Firstly, the online PEM search was conducted at a single point in time using 1 search engine and term, which may not capture the full range of available materials. Search results may vary over time, with different terms (eg, “Meniere’s syndrome”) or browsers (eg, Bing, Safari) yielding different outcomes. Similarly, emulating patient interactions with LLMs is challenging, as priming prompts like “Act as an expert in Meniere’s disease” may alter responses.^48^ Secondly, readability formulas may be affected by medical jargon, which often includes polysyllabic words or terms with many characters, such as “labyrinthectomy” or “endolymphatic.” These formulas also do not account for non-text elements like videos, charts, and images, which can significantly impact the overall comprehension of a text.^49^ Lastly, the DISCERN tool is validated only for treatment-related information and does not assess other disease aspects, such as symptoms or diagnosis, restricting the evaluation to treatment-focused content.

## Conclusion

Both online PEMs and LLM outputs for Meniere’s disease fail to meet the recommended readability levels for the general population and are generally of poor quality and reliability. LLM outputs were more difficult to read, less reliable, and had lower quality as compared to online PEMs, highlighting the importance for physicians to be aware of these limitations when guiding patients. These findings highlight the need for better education materials on Meniere’s disease, emphasizing that both patients and clinicians must understand the limitations of online PEMs and LLMs to improve decision-making and health outcomes.
